# NM23 Is a CP-Binding Protein Involved in Infectious Hypodermal and Hematopoietic Necrosis Virus Infection in Shrimp

**DOI:** 10.3390/ani12050621

**Published:** 2022-03-01

**Authors:** Xiaotong Yin, Xiaoshan Wang, Hui Sun, Rongmei Fei

**Affiliations:** College of Veterinary Medicine, Nanjing Agricultural University, Nanjing 210095, China; xiaotong.yin@remegen.cn (X.Y.); wangxiaoshan0112@163.com (X.W.); 2021807126@stu.njau.edu.cn (H.S.)

**Keywords:** IHHNV, capsid protein, gill membrane proteins, receptors, VOPBA, NM23

## Abstract

**Simple Summary:**

In this study, we aimed to identify the putative host cell receptor for Infectious Hypodermal and Hematopoietic Necrosis Virus (IHHNV) CP(Capsid Protein) in the gill membrane of *LitoPenaeus vannamei*. We established that NM23 is a host cell binding partner for IHHNV CP. Our study is probably the first to address the host cell IHHNV receptor and could provide novel insights into the pathogenesis of IHHNV. We feel that this paper is of interest to the readers of *Animals*.

**Abstract:**

The aim of this study was to identify the putative host cell receptor for Infectious Hypodermal and Hematopoietic Necrosis Virus (IHHNV) CP in the gill membrane of *L. vannamei*. Putative CP binding partners were screened first using a 2-dimensional Virus Overlay Protein Blot Assay (VOPBA) to probe isolated gill membrane proteins using recombinant CP. Putative binding partners were identified using mass spectrometry. A Phage Display Random Dodecapeptide Library was used to screen for dodecapeptides and motifs that bound to CP. Finally, putative binding pairs were confirmed using GST(glutathione-S-transferase) pulldown assays. 2-Dimensional VOPBA identified NM23 as a putative binding partner for IHHNV CP. GST pulldown experiments confirmed the direct interaction of NM23 and IHHNV CP. The phage display library was used to identify six groups of dodecapeptides that bound to CP. From these peptides, three characteristic binding motifs were identified, SW*Y, SKWV, and PQR. Interestingly, the SW*Y motif was also found in NM23. We are the first to implicate NM23 in IHHNV infection and postulate that it may bind to the CP using the SW*Y motif, although this remains to be confirmed.

## 1. Introduction

Infectious Hypodermal and Hematopoietic Necrosis (IHHN), also known as Runt-Deformity Syndrome (RDS), is caused by the IHHN virus (IHHNV) and was identified in breeding shrimp (*LitoPenaeus stylirostris*) in Hawaii, USA in 1981 [1]. The virus is one of the most serious shrimp diseases and has a wide host range. IHHNV is highly pathogenic and is associated with a high degree of mortality in *L. stylirostris*. In contrast, in *L. vannamei*, IHHNV results in chronic infection, slow growth, and lesions in the forehead sword, antenna, and the shell of the cephalothorax and abdomen. The virus infects ectodermal organs, such as the gills epidermis, intestinal epithelial cells, nerve cord, and ganglion, and mesodermal organs, such as the hematopoietic tissue, antennal gland, gonads, lymphoid organs, connective tissue, and striated muscle [2,3]. IHHNV is a non-enveloped, linear, single-stranded DNA virus belonging to *Brevidensovirus Densovirinae Parvoviridae*. IHHNV is the smallest known shrimp virus, reaching only 22 nm in diameter and containing 4.1 kb of DNA [4]. The virus contains three open reading frames (ORFs) and non-coding sequences in two terminals. ORF1 and ORF2 code nonstructural protein, while ORF3 codes the structural capsid protein (CP). The World Organization for Animal Health (OIE) listed IHHNV as a key crustacean virus that must be declared.

Since IHHNV was identified, it has circulated around the world, and many studies have been undertaken including epidemiological surveys and those establishing diagnostic methods, but the pathogenic mechanism(s) of IHHNV infection are still relatively unknown. Viral tropism is determined by the interaction between host cell receptors and viral proteins. Virus Overlay Protein Blot Assay (VOPBA) is a classic method used to identify virus receptors. VOPBA was recently used to identify the host cell receptors for White Spot Syndrome Virus (WSSV) and Yellow Head Virus (YHV). Sritunyalucksana et al. identified PmRab7, a hemocyte membrane receptor, for WSSV [5], and Yan Liang described a role for the F1-ATP synthase beta subunit as a gill membrane receptor in WSSV infection [6]. Regarding YHV, Havanapan et al. identified CrustinPm1 as a viral receptor on *Penaeus monodon* hemocytes [7].

Therefore, we have undertaken the current study to identify the host cell receptor. This study is the first to address the host cell IHHNV receptor and could provide novel insights into the pathogenesis of IHHNV.

## 2. Materials and Methods

### 2.1. Isolation of Shrimp Gill Membrane Proteins

Gill tissue from *L. vannamei* was confirmed to be uninfected by PCR using the OIE recommended primers to detect IHHNV. The gill membrane proteins were prepared using a previously published method [8]. Briefly, the gill tissue (from 10 *L. vannamei*) was homogenized in a glass homogenizer by adding 5 volumes of ice-cold RSB-NP40 (1.5 mM MgCl_2_, 10 mM Tris-HCl, 10 mM NaCl, 1% NP-40, and 2 mM EDTA). Prior to homogenizing the tissue, protease inhibitor cocktail (Amresco, Philadelphia, PA, USA) was added into RSB-NP40. The entire process was conducted on ice. The homogenate was centrifuged at 600 g for 10 min at 4 °C. The supernatant was retained and centrifuged again at 100,000 g for 20 min at 4 °C. The pellet containing the gill membrane proteins was resuspended with 200 uL RSB-NP40. The gill proteins were quantified using a Nanodrop (Thermo, Salem, MA, USA) and stored at −70 °C.

### 2.2. Two-Dimensional VOPBA (2-DE VOPBA)

2-DE VOPBA was used to identify gill proteins that bound to IHHNV. Gill membrane proteins (200 g) were separated simultaneously on two 2-DE gels, in parallel. After the gill membrane proteins were separated by 2-DE, one of the gels was silver stained, and the other was transferred onto a nitrocellulose membrane. As described previously [9,10,11], the nitrocellulose membrane was renatured in four kinds of buffer. The membrane was washed twice in 30 mL of buffer A, B, or C, respectively, for 20 min and then washed quintic in 30 mL of buffer D for 10 min (Buffer A: 20 mM Tris-HCl, 20% isopropanol, pH 8.0. Buffer B: 20 mM Tris-HCl, 4 mM 2-mercaptoethanol, pH 8.0. Buffer C: 20 mM Tris-HCl, 4 mM 2-mercaptoethanol, 6 M guanidine HCl, pH 8.0. Buffer D: 20 mM Tris-HCl, 4 mM 2-mercaptoethanol, 0.03% Tween-20, pH 8.0). The nitrocellulose membrane was then blocked in PBS containing 5% skim milk overnight at 4 °C and then washed in PBST and balance buffer (10 mM Tris-HCl, 5 mM CaCl_2_, 10 mM MgCl_2_, pH 6.5) to equilibrate [10,11]. The membrane was then incubated with 0.8 mg purified CP (prokaryotic expressed) diluted in balance buffer with 0.02% skim milk and 1% Triton X-100 for 2–3 h at 37 °C. After being washed, the nitrocellulose membrane was incubated with rabbit anti-CP polyclonal antibody (1:2000) for 1 h at 37 °C. Finally, the membrane was incubated with goat anti-rabbit IgG-conjugated HRP (1:5000) and developed using the DAB Horseradish Peroxidase Color Development Kit (Beyotime, Shanghai, China). The negative control was a nitrocellulose membrane that was not incubated with CP developed under the same conditions. The nitrocellulose membrane and silver-stained gel were then compared, and the corresponding colored dots on the silver stained gel were extracted and analyzed by MS/MS and the MASCOT program.

### 2.3. Screening Phage Display Random Dodecapeptide Library with CP Protein

Four rounds of screening were conducted using the phage display random dodecapeptide library (New England Biolabs, Salem, MA, USA) according to the manufacturer’s protocol. To optimize performance, the concentration of coating CP protein (100, 80, 70, and 60 µg/mL) and the concentration of input library (2.3 × 10^11^, 2.0 × 10^10^, 1.2 × 10^9^, and 3.0 × 10^8^ pfu/mL) were successively reduced. Meanwhile, the content of Tween-20 in the TBST was successively increased (0.1%, 0.3%, 0.5%, and 0.7%). The eluate from the fourth round was plated on an LB/IPTG/X gal plate, and the resulting single clones were amplified. An ELISA (Enzyme-linked immunosorbent assay) of the fourth-round eluate and single clones was conducted with HRP-M13 monoclonal antibody. DNA from the positive single clones were extracted and sequenced.

### 2.4. Recombination and Expression of NM23 Protein

The total RNA from the gill tissue of *L. vannamei* was extracted and then reverse transcribed into cDNA. Homologous recombination was used to amplify the NM23 gene (Accession Number: DQ907945) using the forward primer 5′-GAATTCCCGGGTCGACTCGAGGTTCGCGAACGCACTTTCAT-3′ (*Xho*I) and reverse primer 5′-GTCACGATGCGGCCGCTCGAGCTCGTAGATCCAGCTCTCGTTGGT-3′ (*Xho*I). The PCR amplicon was cloned into the pGEX-4t-1 vector and transformed into *E. coli* DH5α. The plasmid containing NM23 (pGEX-4t-1-NM23) was verified by digestion and sequencing, and then the vector was transformed into *E. coli* strain BL21 (BL21-pGEX-4t-1-NM23). The NM23 protein was expressed at 37 °C.

### 2.5. 1-DE VOPBA

The basic protocol for VOPBA has been described previously in Section 2.3. The protocol was modified slightly to identify the combination of NM23 and IHHNV CP using one dimensional VOPBA. NM23 and CP expression were induced separately from BL21-pGEX-4t-1 and BL21-pGEX-4t-1-NM23 and then separated by SDS-PAGE. Bound proteins were detected using ECL chemiluminescence

### 2.6. GST Pull-Down Assay

Glutathione Sepharose™ 4B was used to identify the binding interactions between NM23 and IHHNV CP. Protein expressed from BL21-pGEX-4t-1-NM23 containing bacteria was incubated with Glutathione Sepharose™ 4B for 1 h and then centrifuged at 500 g for 5 min. PBS was used to wash (6–8 washes) away the unbound protein. Purified CP was incubated with Glutathione Sepharose™ 4B for 2 h and then centrifuged at 500 g for 5 min and washed 6–8 times. At the end of this assay, the objective protein ingredient was eluted by reduced glutathione. The control groups for the GST pulldown were: GST-NM23 protein alone, GST plus CP, and GST alone. The eluted components were separated by SDS-PAGE and then silver stained. The silver stained band that was similar to CP in size was analyzed by LC-MS/MS.

## 3. Results

### 3.1. NDPK Was Identified by 2-DE VOPBA

**Figure 1:**
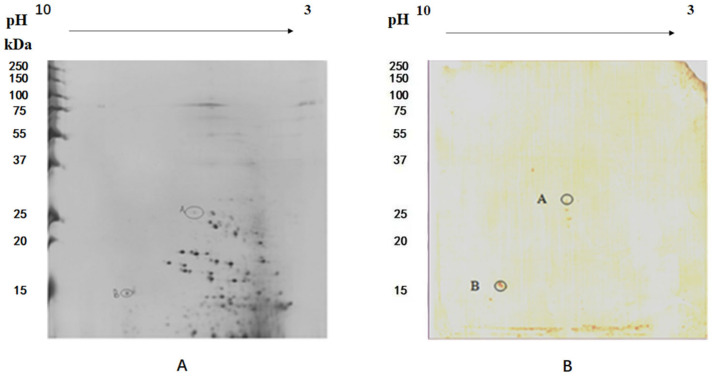
Identification of putative host cell receptors using 2-DE VOPBA. (**A**) 2-DE of gill membrane protein; (**B**) Western-blot of gill membrane proteins detected by IHHNV CP.

Two spots, A (30 kDa) and B (17 kDa), were identified on the nitrocellulose membrane. No binding was detected in the negative control. A and B were extracted from the silver stained 2-DE gel and analyzed by MS + MS/MS (Figure 1). We were unable to identify protein A because there was insufficient sample for MS + MS/MS. Protein B resembled Nucleoside Diphosphate Kinase (NDPK) (Accession Number: ABI93176), the expression product of NM23. The relevant information about NDPK from MS + MS/MS was shown as Table 1. The full length NM23 cDNA is 709 bp, with a 456 bp ORF (55–510 bp) encoding 151 deduced amino acids. The nucleoside diphosphate kinase group I (NDPK-I)-like domain is found between 64–453 bp. The region from 64–468 bp has nucleoside diphosphate kinase activity. The active sites consist of 10 parts (85..87, 205..207, 229..231, 313..315, 331..333, 364..366, 394..396, 403..405, 409..414, 436..438). Five regions are likely to form multimers (97..99, 112..120, 127..129, 136..138, 163..171).

### 3.2. Six Motifs Were Screened Out by Phage Display Random Dodecapeptide Library

We used the scanning Phage Display Random Dodecapeptide Library to determine which NM23 motifs bind to CP. The original titer of the library was determined to be 2.3 × 10^13^ pfu/mL. After four rounds of scanning, the ratio of output/input was progressively increased, and the ratio of wash/output was reduced in a stepwise fashion, which indicated good enrichment (Table 2).

An ELISA using the eluate from the fourth round of screening and select single clones were used to confirm the binding between the isolated dodecapeptides and CP. The absorbance at 492 nm of the fourth round of the eluate by ELISA increased successively from a zero blank (Table 3), indicating that the eluate was enriched for CP binding dodecapeptides. ELISA from the single clones identified 10 positive phage clones from 30 possible single clones (Table 3). A 180 bp PCR amplicon was amplified from the DNA of the positive singe clones.

The dodecapeptides that bound to CP were then sequenced and translated into amino acid sequences by the Bioxm program (Table 4). Duplicate amino acid sequences were identified for peptide 1 and 20; 3, 15, and 23; and 11 and 24. The 10 binding dodecapeptides could be divided into 6 kinds: A (SWSSWVYRDPQT), B (HSFKWLDSPRLR), C (SSFKWLDSPRLR), D (HRSKWVYSDPQR), E (YWSKWVDWHPQR), and F (SSCKWVDWD*AE). There was a single amino acid difference between B (H) and C (S) at position 1. Both of D and E contained the sequences SKWV and PQR, which indicated that these sequences might be characteristic of peptides that bind to IHHNV CP. Interestingly, the C-terminal amino acid sequence of NM23 and A (SWSSWVYRDPQT) contained a similar sequence, SW*Y. Thus, it is plausible that the motif SW*Y is used to bind CP by both group A peptides and NM23.

### 3.3. Prokaryotic Expression of NM23

To further confirm the interaction between NM23 and IHHNV CP, BL21-pGEX-4t-1–NM23 was constructed and expressed. The actual molecular mass of the NM23 protein was 44 kDa, including the molecular mass of the GST tag.

### 3.4. Identification of NM23 by 1-DE VOPBA

**Figure 2:**
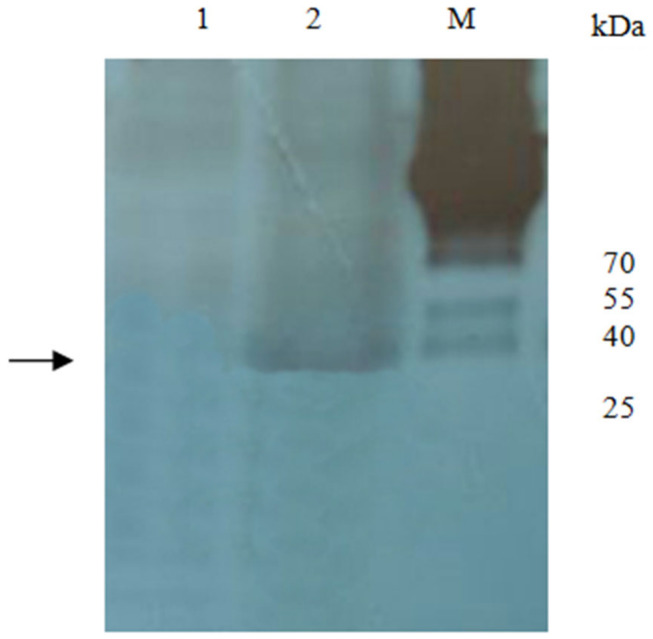
Cetection of NM23 by one-dimensional VOPBA using IHHNY CP. Lane 1. BL21-pGEX-4t-1 + IHHNY Cp; Lane 2. BL21-pGEX-4t-1-NM23 + IHHNV CP; M. Protein marker.

Using 1DE-VOPBA, there was an approximately 40 kDa band consistent with NM23, but, in contrast, there was no GST protein in the vicinity of 26 kDa (Figure 2).

### 3.5. Identification of NM23 by GST PULL-DOWN

**Figure 3:**
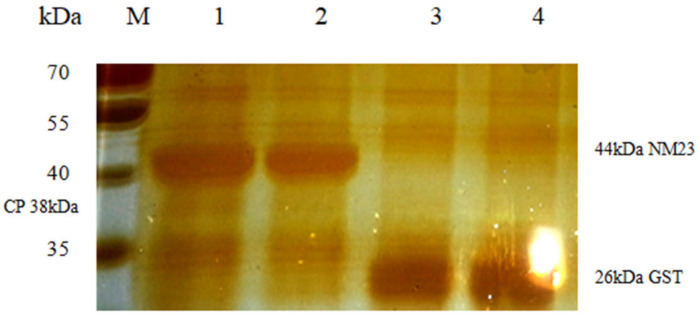
SDS-PAGE analysis of GST PULL-DOWN. Lane 1. GST-NM23 + IHHNV CP; Lane 2. GST-NM23; Lane 3. GST + IHHNY CP; Lane 4. GST; M. Protein marker.

The eluates of the pulldown assay were separated by SDS-PAGE and then silver stained (Figure 3). In Lane 1, but not in Lane 2, there was a band present at approximately 35–40 kDa, which corresponded to the molecular mass of IHHNV CP. No band was detected in Lane 3 at that molecular weight; this eliminated the possibility of binding between GST and CP and indicated that the wash process was adequate. The band in the vicinity of 35–40 kDa was extracted and analyzed by LC-MS/MS (Table 5). With IHHNV CP, NM23 was eluted, indicating an interaction between NM23 and IHHNV CP.

## 4. Discussion

IHHNV has become a serious concern because of its wide range distribution and host range. Shrimp generally do not show severe clinical symptoms after infection with IHHNV [12]. IHHNV-infected *Penaeus monodon* and *P. vannamei* show slow growth, body deformities, and low mortality, so it is also known as Runt-Deformity Syndrome (RDS) [13,14,15,16,17]. There are comparatively few studies that address the pathogenesis of IHHNV; previous work has focused predominately on the epidemiology and diagnostic methods. The first step of virus infection of cells is that the virus binds to receptors on the surface of the cell membrane, and the receptors determine the cell tropism of the virus [18]. Here, we established that NM23 is a host cell binding partner for IHHNV CP.

NM23 is a member of the nucleoside diphosphate kinase (NDPKs) family and is primarily located in the cytoplasm and on the nucleus but can be detected on the cell membrane [19]. NDPKs have various cellular functions, such as altering the dynamics of microtubule polymerization, cell proliferation, development, differentiation and apoptosis, and conditioning G protein signaling pathways [20]. NDPKs could possibly contribute to the pathogenesis of IHHNV in three ways. First, NDPKs are critical for generating GTP, which is needed for microtubule polymerization. NDPKs catalyze the reaction of GDP to GTP and participate in the formation of microtubule spindles. This affects the cytoskeleton and induces cellular movement and viral invasion [21,22]. Therefore, speculatively, there may be a coupling mechanism that induces a cytoskeletal change prompting the endocytosis of virus particles. In fact, NDPK B has been reported to directly interact with G protein coupled receptors (GPCRs) to regulate endocytosis [23]. Secondly, NDPKs have been shown to interact with various proteins such as Pn and Strap, directly and indirectly, to affect the cell proliferation and apoptosis. The binding of NDPK and Pn changes the balance of local nucleotides, which eventually inhibits downstream cell growth [24]. NDPKs and Strap synergistically inhibit the TGF-B signal pathway and regulate cell proliferation [25]. In addition, the combination of NDPKs and Strap likely interact with p53 to enhance cell cycle arrest and p53 mediated apoptosis [26]. Finally, NDPKs control the activity of 3′–5′ exonuclease, which mediates apoptosis through the formation of incisions [27]. Taken together, the evidence from mammalian cells is suggestive that NDPKs not only mediate IHHNV cell invasion but are involved in the cytopathic effect after viral invasion.

The results of the 2DE-VOPBA were confirmed and expanded using a Phage Display Random Dodecapeptide Library to identify dodecapeptides that bound to IHHNV CP. We identified six groups of dodecapeptides that bind to IHHNV CP. Of these, two groups have identical sequences except at position 1. We also identified three putative characteristic binding motifs: SKWV and PQR from the two groups of binding peptides and SW*Y, shared by NM23 and group A of the binding peptides. The VOPBA results were further confirmed using GST-pulldown assays.

Currently, there is minimal research on putative host IHHNV receptors. The lack of research is likely attributable to the lack of a mature shrimp cell line. In this study, traditional VOPBA and the Phage Display Random Dodecapeptide Library were used to identify NM23 as a putative host receptor for IHHNV CP.

Viruses compete for receptors when infecting organisms. Tang et al. [28] found that *Penaeus stylirostris* was first infected with IHHNV and then infected with WSSV, and the mortality of *Penaeus stylirostris* decreased significantly. Melena [29] also did the same experiment on *P. vannamei*, and the results were consistent. It is speculated that IHHNV and WSSV may compete for the same receptor. Subsequent studies showed that Wsv497 and Wsv035 of the White Spot Syndrome Virus (WSSV) could interact with the NM23 protein by VOPBA and co-immunoprecipitation. It is preliminarily speculated that WSSV and IHHNV may compete for the NM23 protein receptor on the shrimp gill cell membrane. This result provides a theoretical basis for future research on the mechanism of competition between the two viruses for cell membrane receptors and shrimp virus protein [30].

## 5. Conclusions

This study mainly focus on the gill cell membrane receptors of the IHHNV capsid protein. To scan out the receptors, VOPBA, pulldown, the phage dodecapeptides display library were used. This study could provide novel insights into the pathogenesis of IHHNV.

## Figures and Tables

**Table 1:** Mass spectrometry identification of protein B compared to the best-matched database.

Protein Name	Accession No.	Protein MW	Protein PI	Pep.Count	Protein Score
NM23protein (*LitoPenaeus vannamei*)	gi:115291340	17,153.7	6.74	4	101
Peptide Information					
Calc.Mass	Observ. Mass	Start Seq.	End Seq.	Seqence	Lon Score
867.4756	867.4084	31	38	GFKLAGMK	
998.6356	998.6215	18	26	GLIGEIKR	
1052.483	1052.4567	105	113	GDFCIEVGR	33
1330.7478	1330.7241	6	17	TRIAVKPDGVQR	50

**Table 2:** Selective enrichment of phage peptides by screening for binding to IHHNV CP.

	1st Screening (pfu/mL)	2nd Screening (pfu/mL)	3rd Screening (pfu/mL)	4th Screening (pfu/mL)
Input	2.3 × 10^11^	2.0 × 10^10^	1.2 × 10^9^	3.0 × 10^8^
Wash	1.4 × 10^11^	1.2 × 10^10^	1.0 × 10^9^	2.4 × 10^8^
Output	5.0 × 10^6^	1.4 × 10^6^	1.0 × 10^6^	3.1 × 10^4^
Output/Input	2.2 × 10^−5^	7.0 × 10^−5^	8.3 × 10^−5^	1.0 × 10^−4^
Wash/Output	2.8 × 10^6^	8.6 × 10^5^	1.0 × 10^4^	8.0 × 10^3^
Amplification	2.0 × 10^12^	1.2 × 10^13^	3.0 × 10^12^	2.0 × 10^12^

**Table 3:** Assessing specific binding of the eluted dodecapeptides/cloned phage peptides to IHHNV CP by ELISA.

Phage Peptides	IHHNV CP	BSA
Elution 1	0.129	0.045
Elution 2	0.134	0.058
Elution 3	0.184	0.056
Elution 4	2.256	0.090
M 13KE	0.060	0.045
1	0.126	0.056
3	0.113	0.057
11	0.219	0.082
15	0.123	0.068
20	0.181	0.059
23	0.105	0.050
24	0.156	0.054
27	0.181	0.084
28	0.139	0.052
29	0.175	0.075
M13KE	0.035	0.200

**Table 4:** Insert sequences from each of the cloned phages.

Cloned Phage No.	Amino Acid Sequence
1	SWSSWVYRDPQT
3	HSFKWLDSPRLR
11	SSFKWLDSPRLR
15	HSFKWLDSPRLR
20	SWSSWVYRDPQT
23	HSFKWLDSPRLR
24	SSFKWLDSPRLR
27	HRSKWVYSDPQR
28	YWSKWVDWHPQR
29	SSCKWVDWD*AE

*: TAG. It was random library, so it was possible and reasonable to have any codon.

**Table 5:** Analysis of NM23 GST pulldown assays by the LC-MS/MS and MASCOT program.

Protein	NCBI	Databasenumber	Match Peptides	Unique Peptides	Sequence Coverage (%)	Mol.Weight (kDa)	Match Peptides Number
Viralcapsid protein	ACF32342.1	B4YSS4	6	1	18.8	37.623	3; 6; 8; 11; 37; 45
Capsid protein	AFR11650.1	J7LD90	8	3	26.4	37.397	3; 6; 7; 8; 11; 39; 45; 52

## Data Availability

The data used are available from the authors upon request.
